# A case of improvement of clozapine-induced low leukocyte counts by adenine, cepharanthin and ninjin-yoei-to in a patient with treatment-resistant schizophrenia

**DOI:** 10.1186/s40780-021-00227-6

**Published:** 2021-12-02

**Authors:** Shintaro Kamei, Ryota Tanaka, Hirofumi Hirakawa, Motoshi Iwao, Rikako Kawanaka, Ryosuke Tatsuta, Takeshi Terao, Hiroki Itoh

**Affiliations:** 1 Department of Clinical Pharmacy, Oita University Hospital, Yufu-shi, Oita 879-5593 Japan; 2 Department of Neuropsychiatry, Oita University Faculty of Medicine, Yufu-shi, Oita 879-5593 Japan

**Keywords:** Treatment-resistant schizophrenia, Clozapine, Leukopenia, Ninjin-yoei-to

## Abstract

**Background:**

Although clozapine is the optimal drug for patients with treatment-resistant schizophrenia, the drug has harmful adverse effects such as leukopenia. Adenine and cepharanthine are known to be effective for radiation- or drug-induced leukopenia. Furthermore, ninjin-yoei-to, a Chinese herbal medicine, augments the production of granulocyte-macrophage colony-stimulating factor. Thus, these drugs may be useful for clozapine-induced leukopenia.

**Case presentation:**

A 21 years-old woman with schizophrenia was hospitalized for initiation of clozapine treatment. Despite concomitant use of adenine, cepharanthine, and lithium carbonate having activities of increasing leukocytes, a decrease in leukocyte counts occurred after the initiation of clozapine. Additional administration of ninjin-yoei-to increased leukocyte counts, which prevented the development of leukopenia.

**Conclusions:**

This is the first case that concomitant use of adenine, cepharanthin, and ninjin-yoei-to exhibited the effectiveness of reversing the decrease in leukocytes caused by clozapine. Monitoring leukocyte counts and preventing leukopenia are essential for successful treatment with clozapine for refractory schizophrenia. These medicines may be a potential option for preventing clozapine-induced leukopenia.

## Background

Schizophrenia is a psychiatric disorder with positive symptoms such as hallucination and delusion; negative symptoms such as apathy, anhedonia and hypoactivity; and cognitive impairment as a prominent symptom. The disease occurs frequently between puberty and adulthood, and exhibits a high prevalence and recurrence rate [1]. Pharmacotherapy with serotonin/dopamine D_2_ receptor antagonist, dopamine D_2_ receptor partial agonist and multi-acting receptor targeted-antipsychotic is the core treatment. However, approximately 20 to 30% of patients with schizophrenia exhibit drug resistance despite receiving multiple antipsychotics at adequate doses for sufficient durations [2].

Clozapine is the optimal drug for patients with treatment-resistant schizophrenia. Previous reports demonstrated that relative to other antipsychotics, clozapine exerts a superior therapeutic effect and a lower incidence of extrapyramidal disorder that is a typical adverse effect of antipsychotics [3, 4]. However, some doctors are reluctant to prescribe clozapine because the drug has harmful adverse effects such as granulocytopenia; especially, rare but potentially life-threatening agranulocytosis puts a relatively high burden on the patient when it develops [5]. Clozapine-induced agranulocytosis is defined as a condition in which the number of neutrophils in the blood is less than 500/μL, which is known to have a mortality rate of 2–4% due to a severe state of reduced resistance to infectious diseases [6]. Therefore, early detection and management of reduced granulocytes are required to continue treatment by clozapine. In Japan, it is obligatory to monitor neutrophil and leukocyte counts by frequent blood tests according to the procedure of Clozaril Patient Monitoring Service, in order to detect a reduction in these counts at an early stage [7]. Furthermore, clozapine must be discontinued when the number of neutrophils decreases to less than 1500/μL or leukocytes to less than 3000/μL, to prevent the onset of agranulocytosis.

Some drugs having activities of increasing leukocytes and neutrophils have been developed. Adenine and cepharanthine are known to be effective for radiation- or drug-induced leukopenia [8–10]. Lithium carbonate, an antimanic drug, stabilizes a person’s mood and is used as an add-on treatment with antipsychotics for schizophrenia. In previous reports, the numbers of leukocytes and neutrophils increased significantly in patients with bipolar disorder who received lithium carbonate [11–13]. Furthermore, ren-shen-yang-rong-tang (Japanese name: Ninjin-yoei-to, NYT), a traditional Chinese herbal medicine, augments the production of granulocyte-macrophage colony-stimulating factor (G-CSF) [14] and exerts a protective effect for cyclophosphamide- or 5-fluorouracil-induced leukopenia [15].

We report a patient with treatment-resistant schizophrenia whose leukocyte counts decreased by clozapine treatment despite concomitant use of adenine, cepharanthine and lithium carbonate, and additional administration of NYT succeeded to prevent the development of leukocytopenia. This case report was approved by the ethics committee of Oita University (approval number: 2054), and written informed consent for publication of this report was obtained from the patient.

## Case presentation

**Fig. 1:**
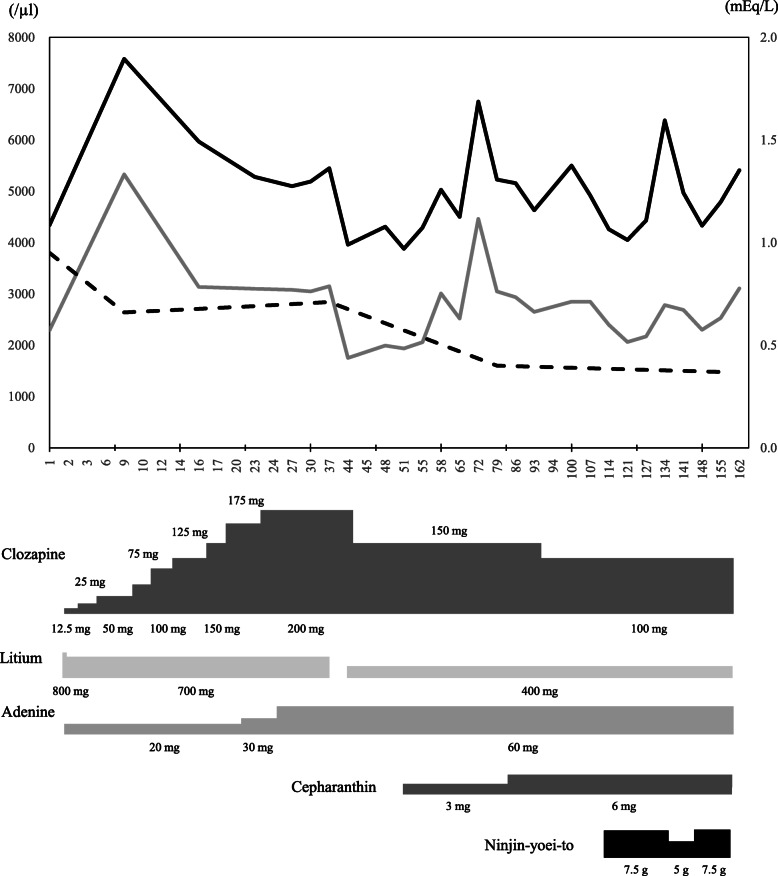
Clinical course after the initiation of clozapine therapy during hospitalization. Black solid line represents leukocyte counts (left vertical axis), gray solid line represents neutrophil counts (left vertical axis), and dotted line represents lithium concentration (right vertical axis)

A 21-year-old woman with schizophrenia was hospitalized in the psychiatry ward at Oita University Hospital for initiation of clozapine treatment. Symptoms such as auditory hallucination, loosening of association, delusion of control, thought insertion, loss of motivation, and decreased comprehension were observed at admission. As a physical symptom, hypersalivation was conspicuous, and she was wiping her mouth repeatedly. Moreover, hand tremors and constipation were also reported. The drugs used on admission were olanzapine (20 mg/day), quetiapine (100 mg/day), etizolam (1.5 mg/day), biperiden (2 mg/day) and flunitrazepam (2 mg/day). The leukocyte and neutrophil counts were 4720/μL and 1987/μL, respectively, at the time of admission. Neutrophil count was below the criterion for starting clozapine treatment (white blood cells ≥4000/μL, neutrophils ≥2000/μL) in Japan. Administration of lithium carbonate (600 mg/day) was initiated from day − 20 in anticipation of a leukocyte increasing effect, and the dose of this drug was increased to 800 mg/day after dose adjustment by therapeutic drug monitoring (0.6–0.7 mEq/L). Since both leukocyte and neutrophil counts fulfilled the starting criteria (we defined this day as day 1) (Table 1), administration of clozapine was initiated from 12.5 mg/day on day 2. Figure 1 illustrates the clinical course after initiation of clozapine therapy. The dose of clozapine was up-titrated gradually according to the package insert in Japan. Adenine was used concomitantly aiming to increase leukocyte counts since these counts were low at the initiation of clozapine. Although leukocyte counts increased significantly by concomitant use of adenine, the counts tended to decrease from day 9 along with a gradual increase of clozapine dose. Since psychological symptoms were not exacerbated, the doses of olanzapine and quetiapine were reduced gradually and discontinued on day 23 and day 30, respectively. With the discontinuation of these drugs, hand tremors and hypersalivation improved significantly, and hence biperiden was discontinued. Constipation also improved with the discontinuation of biperiden. On day 44, since the leukocyte and neutrophil counts decreased to 3960/μL and 1750/μL, respectively, the dose of clozapine was reduced from 200 mg/day to 150 mg/day. Furthermore, cepharanthine was added to promote the increase in leukocyte counts. This drug was used with approval for off-label use in Oita University Hospital, and after obtaining written informed consent from the patient and her families. After the initiation of cepharanthine, the leukocyte counts increased gradually reaching a peak on day 72, and thereafter declined gradually. Despite decreasing the dose of clozapine to 100 mg/day, leukocyte count decreased to 4260/μL and neutrophil count to 2398/μL on day 114. On the other hand, decreasing the dose of clozapine did not cause exacerbation of the psychiatric symptoms of schizophrenia.

**Table 1:** Laboratory findings and clinical parameters on day 1

Item	Value	Item	Value
Height (cm)	156.5	Uric acid (mg/dL)	7.72
Weight (kg)	66.6	Blood urea nitrogen (mg/dL)	11.2
Body temperature (°C)	35.6	Serum creatinine (mg/dL)	0.55
Systolic blood pressure (mmHg)	111	Serum sodium (mmol/L)	138.9
Diastolic blood pressure (mmHg)	79	Serum potassium (mmol/L)	3.90
Pulse rate (bpm)	96	White blood cell count (× 10^3^/μL)	4.34
Blood glucose (mg/dL)	85.0	Neutrophil cell count (× 10^3^/μL)	2.29
Hemoglobin A1c (%)	5.6	Red blood cell count (× 10^6^/μL)	4.36
Serum albumin (g/dL)	4.33	Hemoglobin (g/dL)	13.0
Aspartate transaminase (U/L)	22.1	Hematocrit (%)	38.4
Alanine transaminase (U/L)	27.1	Platelet (*10^3^/μL)	350.0
Alkaline phosphatase (U/L)	244	Total cholesterol (mg/dL)	182.0
γ-glutamyl transpeptidase (U/L)	44.6	Triglyceride (mg/dL)	172.0
Total bilirubin (mg/dL)	0.49	HDL cholesterol (mg/dL)	40.5
		LDL cholesterol (mg/dL)	107.1

*HDL* High-density lipoprotein, *LDL* Low-density lipoprotein

**Fig. 2:**
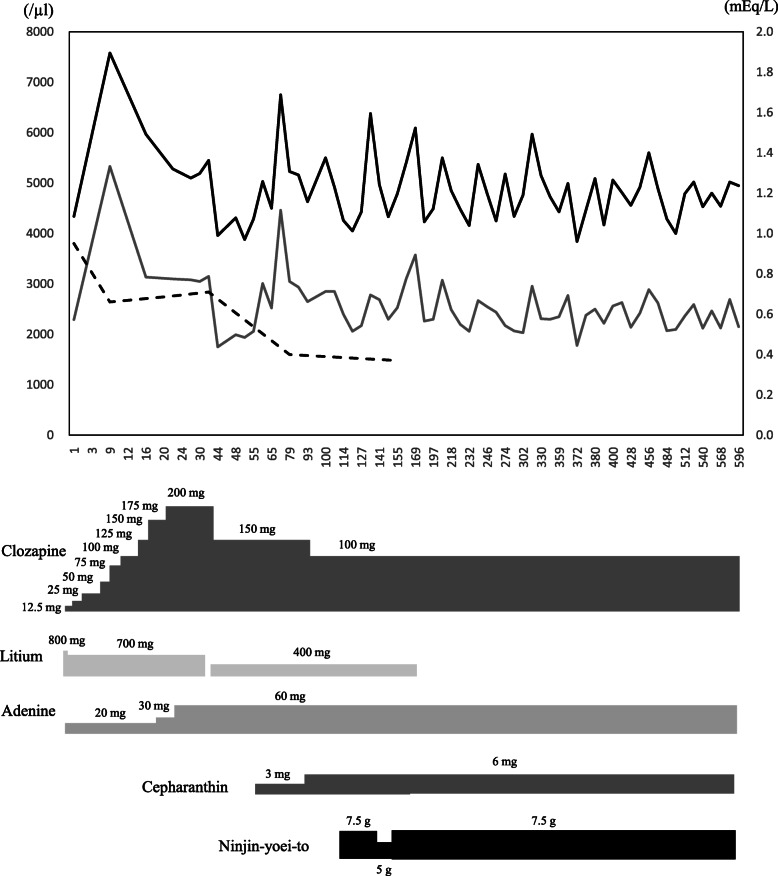
Clinical course from the initiation of clozapine therapy to Day 596. Black solid line represents leukocyte counts (left vertical axis), gray solid line represents neutrophil counts (left vertical axis), and dotted line represents lithium concentration (right vertical axis)

To improve the low leukocyte counts, the patient was additionally given NYT (2.5 g three times daily), a Chinese herbal medicine from day 114. Leukocyte and neutrophil counts remained low at around 4000/μL and 2000/μL, respectively, until day 126, but increased to 6380/μL and 2781/μL on day 133, 2 weeks after the initiation of NYT. However, since the patient complained that this medicine was “bitter and difficult to swallow”, the dosing schedule was changed to 2 times daily from day 133. On day 148, leukocyte and neutrophil counts decreased to 4330/μL and 2299/μL, and the dosage regimen of NYT was returned to 3 times daily. After changing the dosage regimen, both white blood cell count and neutrophil count increased. Thereafter, she was discharged from the hospital on day 162 because her psychiatric symptoms were stable. Even after discharge, leukocyte and neutrophil counts did not fall below the criteria for discontinuation of clozapine, and treatment was continued for 600 days or more (Fig. 2).

## Discussion and conclusions

The patient had low leukocyte counts prior to the initiation of clozapine, since she was taking psychotropic drugs such as olanzapine [16] and quetiapine [17] that are known to induce leukopenia. As mentioned in Introduction, clozapine must be discontinued when the number of neutrophils or leukocytes decreases to less than 1500/μL or 3000/μL to prevent the onset of agranulocytosis. Since lithium carbonate [11–13] and adenine [8, 9] have been reported to elevate leukocyte counts, we used these drugs concomitantly with clozapine. However, although no significant leukopenia was found at low doses of clozapine in the early stages of treatment, leukocyte count decreased to less than 3000/μL and neutrophil count to less than 1500/μL as the dose was increased gradually to 200 mg/day. We judged that lithium carbonate and adenine alone could not prevent leukopenia and added cepharanthine, which is known to be effective for radiotherapy-induced leukopenia [10]. The drug was temporarily effective, but both white blood cell count and neutrophil count gradually decreased. Therefore, we further added NYT, which was reported to increase the leukocyte count [15]. Consequently, an increase in leukocyte count was obtained, and treatment was enabled to continue for 600 days or more by the concomitant use of NYT, adenine, and cepharanthin.

Some Chinese herbal medicines have been reported to have immunity enhancing actions, and the representative agents are hochu-ekki-to (HET), juzen-taiho-to (JTT), and NYT. HET exhibits an immunity-enhancing action by activating Th1 and Th2 cells [18]. Experimental studies suggest that JTT increases the number of hematopoietic stem cells [19]. NYT has the activity of augmenting the production of G-CSF [14]. Based on these previous reports, we selected JTT and NYT, which are especially active in increasing leukocytes, as candidates. NYT consists of twelve herbal ingredients and contains more *Angelica sinensis* compared to JTT, which can suppress the cytotoxicity of cyclophosphamide on hematopoietic cells [20]. Therefore, we selected NYT for this patient, consequently confirming a significant increase in leukocyte counts. NYT is commonly prescribed to compensate for dual deficiency of qi and blood, and has also an immunopharmacological feature of restoring immunocompetence by promoting hematopoiesis in immunosuppressive states such as reduced myelopoiesis capability. Moreover, NYT not only promotes the recovery of leukocyte counts via increasing G-CSF, but also promotes the recovery of erythroid progenitor cells and platelets, which cannot be recovered by G-CSF. Hence, the mechanism of action is considered to be through acting on stromal cells that form the hematopoietic microenvironment, rather than direct induction of stem cells [21]. The decrease in leukocyte count in this patient was improved probably by the above-mentioned hematopoietic effect.

This is the first case that concomitant use of adenine, cepharanthin, and NYT, a Chinese herbal medicine, exhibited the effectiveness of reversing the decrease in leukocytes caused by clozapine. Monitoring leukocyte and neutrophil counts and preventing leukopenia and agranulocytosis are essential for successful treatment with clozapine for refractory schizophrenia. NYT, in addition to adenine and cepharanthin may be a potential preventive option. However, since there is only one case and the influence of the reduced dose of clozapine cannot be completely ruled out, adding more cases and performing detailed analyses will be necessary to verify the present finding.

## Data Availability

The data that support the findings of this study are available from the corresponding author upon reasonable request.

## Abbreviations

NYT: Ninjin-yoei-to
G-CSF: Granulocyte-macrophage colony-stimulating factor
HET: Hochu-ekki-to
JTT: Juzen-taiho-to
